# Dietary Adequacy and Its Associated Factors Among Adolescents in a Rural District of Sindh

**DOI:** 10.3390/nu18152438

**Published:** 2026-07-26

**Authors:** Tansheet Jawad, Naureen Rehman, Muzna Hashmi, Arjumand Rizvi, Zahra Ali Padhani, Saleema Gulzar, Hira Farooq, Rasool Bux, Imran Ahmed Chauhadry, Jai K. Das

**Affiliations:** 1 Department of Community Health Sciences, Aga Khan University, Karachi 74800, Pakistan; 2 Institute for Global Health and Development, Aga Khan University, Karachi 74800, Pakistan; noureen.rehman@aku.edu (N.R.); muzna.hashmi2@aku.edu (M.H.); rasool.bux@aku.edu (R.B.); 3 School of Public Health, Faculty of Health and Medical Sciences, University of Adelaide, Adelaide, SA 5005, Australia; 4 School of Nursing and Midwifery (SoNaM), Aga Khan University, Karachi 74800, Pakistan; 5 Department of Pediatrics and Child Health, Aga Khan University, Karachi 74800, Pakistan; hira.farooq@aku.edu (H.F.);; 6 Centre of Excellence in Women and Child Health, Aga Khan University, Karachi 74800, Pakistan

**Keywords:** macronutrients, micronutrients, diet, adolescents, food, nutrition

## Abstract

**Background**: Adolescents in low- and middle-income countries face a disproportionate burden of dietary inadequacy, yet evidence on the determinants of overall diet quality remains scarce. This study assessed the prevalence of macro- and micronutrient inadequate intake and identified factors associated with dietary adequacy among adolescents in rural Pakistan. **Methods**: A cross-sectional survey was conducted among adolescents using multistage cluster sampling. Dietary intake was assessed through a single 24-h dietary recall; nutrient adequacy was determined using age-and sex-specific Dietary Reference Intake (DRI) standards, including Estimated Average Requirements (EARs), Adequate Intakes (AIs), Estimated Energy Requirements (EERs), and Acceptable Macronutrient Distribution Ranges (AMDRs), as appropriate. Overall dietary adequacy was quantified using the Mean Adequacy Ratio (MAR), calculated as the unweighted mean of nutrient adequacy ratios for fourteen nutrients, including protein, fiber, calcium, iron, zinc, thiamin, riboflavin, niacin, vitamins A, B6, C, D, E, and folate, scaled from 0 to 100. Multivariable linear regression incorporating survey weights and clustering was used to identify independent predictors of MAR using STATA. **Results**: A total of 1132 adolescents were included. Most of the energy was derived from carbohydrates, and a high proportion of adolescents had energy intakes below age- and sex-specific Estimated Energy Requirement (EER) values, while very high levels of inadequacies were observed for protein, fiber, calcium, vitamin D, and vitamin A. The mean MAR score was 54.04 ± 16.51, indicating overall poor dietary adequacy. In multivariable analysis, male sex (β = 3.26; 95% CI: 1.76, 4.75), belonging to the richest wealth quintile (β = 5.81; 95% CI: 1.76, 9.86), always eating between meals (β = 6.70; 95% CI: 3.93, 9.47), and use of bar soap for handwashing (β = 4.97; 95% CI: 2.00, 7.94) were significantly associated with higher dietary adequacy. Adolescents who were underweight (β = −2.40; 95% CI: −4.81, −0.11) and those with moderate physical activity levels (β = −3.96; 95% CI: −7.62, −0.29) had significantly lower dietary adequacy scores. **Conclusions**: Dietary inadequacy among rural adolescents in Pakistan was highly prevalent and was associated with socioeconomic, behavioral, and nutritional factors. These findings highlight the need for interventions that address food insecurity, promote equitable food distribution, and strengthen nutrition education to support adolescent health and nutrition. However, these findings should be interpreted in light of the cross-sectional design and the use of a single 24-h dietary recall, which may not reflect habitual dietary intake.

## 1. Introduction

Nutrition is the cornerstone of human health and development through every stage of life [1]. Adolescence represents a critical life stage characterized by rapid physical, cognitive, and emotional changes [2], necessitating elevated needs for both macro- and micronutrients [2,3]. Adolescents aged 10–19 years make up the largest generation of young people in history, and 90% of these live in low- and middle-income countries (LMICs) [4].

Malnutrition remains a major cause of morbidity and mortality among children and adolescents worldwide [1]. Nutritional inadequacy refers to habitual nutrient intake below requirements and may, if persistent, progress to nutritional deficiency, a physiological state characterized by depleted nutrient stores and impaired biochemical or functional status [5]. Micronutrient deficiencies can lead to both severe health issues and subtle decline in energy and cognitive function, ultimately reducing educational attainment, productivity, and disease resistance [6]. Poverty and food insecurity among adolescents compromise the nutritional foundation essential for lifelong health and productivity. In LMICs, adolescents’ shift from traditional diets to more processed, energy-dense, and nutrient-poor foods [7] has led to a triple burden of malnutrition encompassing undernutrition, obesity, and micronutrient deficiencies [6].

Globally, over 340 million adolescents are experiencing undernutrition, and an increasing number are facing overweight and obesity [8]. More than two-thirds of global adolescent deaths occur in low- and middle-income countries (LMICs), with approximately 26% occurring in the South-East Asia Region [1]. According to the National Nutrition Survey (2018) in Pakistan, an estimated 38% of adolescents are underweight, while 17.8% of boys and 16.8% of girls are overweight [2,9]. This growing burden of excess weight is projected to continue, with approximately 5.4 million Pakistani school-aged children expected to be obese by 2030 [3]. In the Global Food Security Index (GFSI) for 2019, Pakistan has been classified as one of the countries at high risk for Food Insecurity (FI) [10], affecting approximately 48% of Pakistan’s population on average, with higher levels of food insecurity observed in rural areas (60.6%) compared to 52.4% in urban areas [11]. Adolescents’ diets are commonly deficient in fruits and vegetables while high in energy-dense, nutritionally deficient foods, such as sugary and salty items, sweetened drinks, and fast food [12]. Poor diets, early marriages, socio-economic disparities, and limited access to nutritious food and healthcare further compound significant nutrition challenges, particularly in rural areas like Tando Mohammad Khan (TMK), where access to healthcare and nutrition education is limited [11,13,14].

Although adolescents account for 24% of Pakistan’s population [8], there is a scarcity of data regarding dietary adequacy estimates among adolescents and the associated socio-demographic factors. While adolescents’ unique nutritional needs are frequently overlooked, this study addresses a critical evidence gap, exploring rural adolescents’ dietary intake, nutritional status, and the associated factors. The objective of this study is to assess dietary intake and its adequacy among adolescents in the rural district of Tando Mohammad Khan (TMK) in Pakistan and to assess the factors associated with dietary adequacy among adolescents in TMK.

## 2. Method

### 2.1. Study Setting

This study was conducted in the TMK district in the province of Sindh in Pakistan. TMK covers an area of 1423 km^2^ and has a population of approximately 677,098, including 73,247 adolescents, according to the 2017 census [4]. The district is predominantly agricultural, with nearly 70% of the population engaged in farming activities. TMK has 1043 schools, primarily primary-level institutions but with low enrollments, and faces significant healthcare and sanitation challenges [5].

### 2.2. Study Design and Study Population

A community-based cross-sectional study was conducted between 1 March 2021 and 31 May 2021 to assess dietary adequacy among adolescents aged 10–19 years residing in TMK, Pakistan. The primary study has been published and provides detailed information on the study methodology [6].

### 2.3. Sample Size and Sampling Technique

The study sample size was calculated to estimate the prevalence of underweight among adolescents aged 10–19 years with a 95% confidence level, 5% precision, a design effect of 2, and an expected response rate of 90%. Using baseline data from the National Nutrition Survey (NNS 2018) [7], which reported an underweight prevalence of 30.7% in boys and 20.1% in girls, a total of 1304 households were selected for the adolescent survey, with an equal gender distribution (652 males and 652 females).

A multistage cluster sampling approach was employed using the 48 clusters from the CoMIC trial as the primary sampling units [8]. The sample was distributed equally across clusters. Within each cluster, households were selected through systematic random sampling using a predefined sampling interval (k) after a random starting point. In households with more than one eligible adolescent, one participant was selected using the Kish grid method. In cases of non-response or refusal, the next household in the predetermined sequence was approached.

### 2.4. Eligibility Criteria

Adolescents aged 10–19 years who were permanent residents of TMK were eligible for inclusion in the study, and only one adolescent per household was enrolled. Adolescents participating in other intervention trials were excluded.

### 2.5. Dietary Assessment and Nutrient Adequacy

Dietary intake was assessed using a single interviewer-administered 24-h dietary recall conducted by trained data collectors. A standardized multiple-pass recall protocol was employed to improve completeness and reduce recall errors. Information regarding all foods and beverages consumed during the preceding 24 h, including meal timing, ingredients, and portion sizes, was recorded. As a single 24-h recall captures short-term dietary intake, it was used to estimate participants’ intake on the day preceding the interview and does not represent usual dietary intake due to within-person day-to-day variation in food consumption. Repeated dietary recalls were not available; therefore, statistical methods for estimating usual intake distributions, such as the National Cancer Institute (NCI) or Iowa State University (ISU) methods, could not be applied. Nutrient intake was estimated using the MAL-ED Pakistan Food Composition Table, a locally adapted database developed using nutrient information from multiple international sources, including the World Food Dietary Assessment System, USDA National Nutrient Database, NUTTAB, UK Composition of Foods Integrated Database, and the Food Composition Table for Bangladesh [15]. The MAL-ED database served as the primary harmonized nutrient source, with food items standardized to common edible portions and nutrient values before coding and analysis. A trained nutritionist assigned food codes and applied nutrient retention factors to account for cooking-related nutrient losses. Total daily energy, macronutrient, and micronutrient intakes were subsequently calculated for each participant.

Overall dietary adequacy was assessed using the Mean Adequacy Ratio (MAR), a validated composite indicator of nutrient adequacy. Nutrient adequacy was evaluated using age- and sex-specific Dietary Reference Intake (DRI) standards. Estimated Average Requirements (EARs) were applied whenever available, whereas Adequate Intake (AI), Estimated Energy Requirements (EER), and Acceptable Macronutrient Distribution Ranges (AMDR) were used for nutrients lacking EAR recommendations.

Energy adequacy was evaluated using age- and sex-specific Estimated Energy Requirement (EER) values from the Dietary Reference Intake framework as no EAR exists for energy, and participants with reported energy intake below the corresponding EER value were classified as having intake below recommended energy requirements.

Nutrient Adequacy Ratios (NARs) were calculated for fourteen nutrients, including protein, fiber, calcium, iron, zinc, thiamin, riboflavin, niacin, vitamin A, vitamin B6, vitamin C, vitamin D, vitamin E, and folate. These nutrients were selected based on: (i) the availability of age- and sex-specific dietary reference standards necessary for nutrient adequacy assessment, primarily EARs and AI values where EARs were unavailable; (ii) their public health relevance among South Asian adolescents; and (iii) their established roles in growth, skeletal development, immune function, and reproductive maturation. Consistent with previous studies, MAR was calculated using selected nutrients indicative of overall dietary adequacy rather than all nutrients available in food composition databases. Nutrients evaluated using alternative dietary reference frameworks, such as energy (EER) and total fat (AMDR), were not included in MAR calculations because these measures are conceptually distinct from nutrient adequacy ratios and are not routinely incorporated into MAR indices.

Each NAR was calculated as the ratio of observed nutrient intake to the corresponding age- and sex-specific dietary reference value and was truncated at 1.0 to avoid overestimation among participants exceeding recommended intakes [9]. The MAR was calculated as the unweighted mean of all fourteen NARs and multiplied by 100, with higher scores indicating better overall dietary adequacy [16].

### 2.6. Covariates and Measurement Tools

Sociodemographic and household characteristics, including age, sex, educational status, household size, socioeconomic status, marital status, sanitation facilities, and handwashing practices, were collected using a structured household questionnaire adapted from the Pakistan Demographic and Health Survey (PDHS) [4]. Household food insecurity was assessed using the FAO Global Food Insecurity Experience Scale [10], while dietary diversity was measured using the USAID FANTA Household Dietary Diversity Scale [10,11]. Meal patterns were assessed using previously validated epidemiological tools [12,13].

Lifestyle-related variables, including physical activity, sleep patterns, and tobacco use, were assessed using items adapted from the WHO Health Behavior in School-aged Children (HBSC) survey [10] and the Global School-based Student Health Survey (GSHS) [10]. Depression was assessed using Beck’s Depression Inventory [14]. Sleep quality was assessed using a single self-reported item adapted from the Health Behaviour in School-aged Children (HBSC) survey, whereby participants rated their overall sleep quality as very good, fairly good, fairly bad, or very bad. Similar single-item measures have been widely used in adolescent epidemiological studies and have demonstrated acceptable validity for assessing subjective sleep quality. Physical activity was assessed through self-reported moderate-to-vigorous physical activity performed during weekdays and weekends. Total weekly physical activity was calculated by summing the duration of activity across all seven days. Participants were subsequently categorized as inactive (<3 days/week of MVPA), moderately active (3–5 days/week of MVPA), or highly active (>5 days/week of MVPA), based on the reported number of days in which they achieved at least 60 min of MVPA, consistent with the WHO recommendations for children and adolescents.

In addition, anthropometric measurements were conducted at the household level by trained data collectors following standardized procedures. Weight was measured to the nearest 0.1 kg using a calibrated Seca floor scale (model 813; Seca GmbH & Co. KG, Hamburg, Germany), while height was measured to the nearest 0.1 cm using a Seca stadiometer (model 213; Seca GmbH & Co. KG, Hamburg, Germany). Participants were measured without footwear and wearing light clothing.

All anthropometric measurements were obtained in duplicate, and in cases where discrepancies exceeded 0.5 kg for weight or 1.0 cm for height, a third measurement was performed by the field supervisor.

### 2.7. Data Collection and Quality Assurance

Data were collected using handheld electronic devices (Samsung tablets operating on Android 5.1) by trained field staff following six days of standardized training on study procedures, questionnaire administration, and data management protocols. Household demographic and socioeconomic information was obtained from the mother or caregiver after informed consent. Built-in range checks, consistency checks, and skip patterns were incorporated into the electronic data collection system to minimize entry errors. Data were synchronized daily and uploaded to the university server for quality monitoring. Daily quality reports were generated by the data management unit, and discrepancies were communicated to field teams for verification and correction. All collected data were encrypted, anonymized, and stored securely.

### 2.8. Statistical Analysis

All statistical analyses were conducted using Stata version 19.0 (StataCorp, College Station, TX, USA). Descriptive statistics were presented as means and standard deviations (SDs) for continuous variables and weighted frequencies and percentages for categorical variables. Nutrient variables were screened for implausible values using percentile distributions, skewness, and kurtosis, supplemented by histograms and boxplots. Values disproportionately exceeding what is biologically achievable through dietary intake for the relevant age group were considered implausible, likely reflecting portion-size entry or food-coding errors in the 24-h dietary recall, and were treated as missing rather than imputed. All descriptive statistics and nutrient adequacy classifications were computed using cleaned variables, with valid sample sizes reported alongside each estimate. Nutrient Adequacy Ratios (NARs) were calculated by dividing individual nutrient intakes by the corresponding age- and sex-specific Estimated Average Requirement (EAR) or Adequate Intake (AI), as appropriate, with values truncated at 1.0 to avoid overestimation of overall adequacy. The Mean Adequacy Ratio (MAR) was calculated as the arithmetic mean of the truncated NARs across the selected micronutrients. Sample weights accounting for the multistage cluster sampling design were incorporated into all analyses using the svy command in Stata. Missing data were assessed for a Missing Completely at Random (MCAR) pattern and were minimal across key study variables.

Univariable survey-weighted linear regression analyses were initially performed to identify potential factors associated with dietary adequacy (MAR). Variables were chosen based on biological plausibility and prior evidence and were only included in the multivariable model if *p* < 0.25. Candidate variables were then evaluated iteratively in the multivariable model, and the final model was based on their independent contribution while maintaining overall model fit and parsimony. The final survey-weighted multivariable linear regression model adjusted for age group, sex, wealth quintile, father’s education, body mass index (BMI), physical activity, eating between meals, and handwashing material. Multicollinearity among covariates was assessed using the variance inflation factor (VIF). Because regression diagnostic procedures are not available following survey-weighted estimation in Stata, an equivalent ordinary least squares (OLS) regression model was fitted to the same analytical sample solely for diagnostic purposes. Residual normality was evaluated using histograms, normal quantile–quantile (Q–Q) plots, and probability–probability (P–P) plots of standardized residuals. Homoscedasticity was assessed using residual-versus-fitted plots and the Breusch–Pagan/Cook–Weisberg test, while influential observations were evaluated using Cook’s distance and leverage statistics (Supplementary Figure S1). Findings are reported as adjusted regression coefficients (β) with corresponding 95% confidence intervals (CIs), and statistical significance was defined as a two-sided *p* < 0.05. The data used for this study are available in the Supplementary Materials. 

### 2.9. Ethical Considerations

Ethical approval for the study was obtained from the Aga Khan University Ethical Review Committee (Ref: 2021-5943-16892). Written informed consent was obtained from parents or legal guardians, along with assent from adolescents aged 10 to <18 years. Adolescents aged 18–19 years provided written informed consent independently. Participants were informed of their right to refuse participation or withdraw from the study at any stage without any consequences.

## 3. Results

### 3.1. Participants’ Socio-Demographics and Household Characteristics

**Figure 1:**
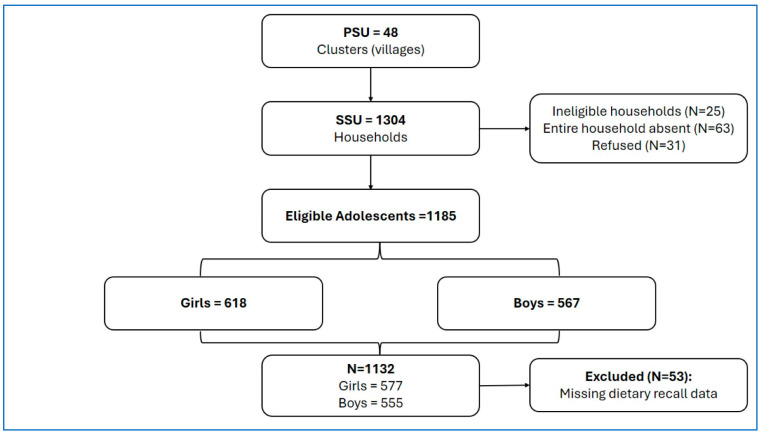
Sampling and participant selection flowchart.

Of the 1304 households selected through systematic sampling, 119 were excluded because the households were locked (n = 63), they refused participation (n = 31), or they did not have an adolescent aged 10–19 years (n = 25), and a further 53 adolescents were excluded because of missing 24-h dietary recall data, resulting in a final analytical sample of 1132 adolescents (577 girls and 555 boys) (Figure 1).

The study included 1132 adolescents aged 10–19 years, with 36.2% aged 13–15 years, followed by 32.8% aged 16–19 years and 31.0% aged 10–12 years. The gender distribution was nearly equal, with 51.0% females and 49.0% males, while most participants belonged to households with more than five family members (64.3%). A large proportion of households had unimproved sanitation facilities (72.1%), although water was available at handwashing places in 92.5% of households, and soap was the most used handwashing material (64.9%). Educational attainment was generally low, with 55.7% of fathers and 88.8% of mothers uneducated, while 62.8% of adolescents were out of school. Food insecurity was highly prevalent, affecting 86.2% of participants, while only 13.8% were food secure. Anthropometric assessment showed that 18.3% of adolescents were underweight and 4.0% were overweight or obese. Additionally, 35.4% of participants were involved in child labor, and 24.1% reported working four or more hours daily. Regarding lifestyle factors, 41.7% reported eating between meals sometimes, while 12.0% engaged in vigorous physical activity (>5 days/week). Overall, 10.7% of adolescents screened positive for depression, and 72.4% reported good sleep quality (Table 1).

### 3.2. Macro- and Micronutrient Intake Among Adolescents

Energy and macronutrient dietary inadequacy were highly prevalent among the study participants. Nearly all adolescents (96.6%) reported energy intakes below age- and sex-specific Estimated Energy Requirement (EER) values. Adequate carbohydrate, protein, and total fat intake was observed among 98.8%, 66.1%, and 70.9% of participants, respectively. Only 17.0% of participants met the Adequate Intake (AI) recommendation for dietary fiber. Micronutrient inadequacy based on Estimated Average Requirements (EARs) was widespread, with particularly high proportions of inadequate intake for calcium (99.4%), vitamin D (99.0%), vitamins A (98.6%) and E (98.3%), folate (94.5%), vitamin C (90.0%), phosphorus (87.3%), magnesium (85.2%), zinc (83.5%), vitamin B6 (66.4%), niacin (35.9%), thiamin (30.5%), riboflavin (29.9%), and iron (33.1%). For nutrients with AI recommendations, only 1.4% and 16.3% of participants met the AI for potassium and sodium, respectively (Table 2).

**Figure 2:**
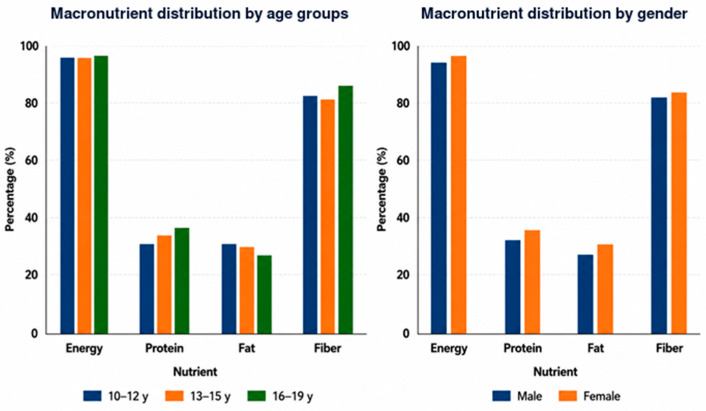
Prevalence of inadequate macronutrient intake by age group and gender.

**Figure 3:**
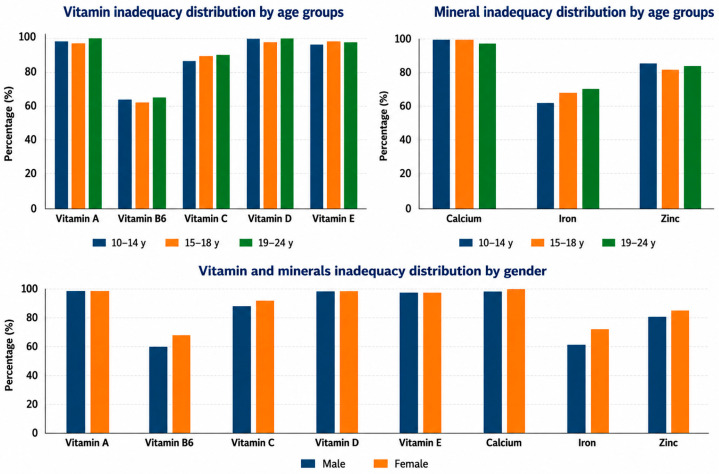
Prevalence of inadequate micronutrient intake by age group and gender.

Overall, inadequate nutrient intake was highly prevalent across all age groups and both sexes (Figure 2 and Figure 3). Among macronutrients, inadequate energy intake remained consistently high across age groups, while protein and fat inadequacy were comparatively lower.

Females had a higher inadequacy of iron, vitamin B6, vitamin C, and zinc compared to males, whereas age-related differences were generally small, with only a modest increase in iron inadequacy observed among older participants.

The overall Mean Adequacy Ratio (MAR) of 54.04 ± 16.51 indicates suboptimal overall nutrient adequacy among adolescents, suggesting inadequate intake of several essential nutrients across the study population. In univariable linear regression analysis, adolescents aged 13–15 years had lower MAR scores compared to those aged 10–12 years (β = −2.7, 95% CI (−4.7, −0.7)), while male adolescents had higher MAR scores than females (β = 3.0, 95% CI (1.1, 4.9)). Adolescents belonging to middle (β = 4.1, 95% CI (0.6, 7.5)) and richest wealth quintiles (β = 11.9, 95% CI (8.5, 15.3)) demonstrated higher dietary adequacy compared to the poorest households. Improved sanitation facilities (β = 4.2, 95% CI (2.0, 6.3)), secondary paternal education (β = 5.0, 95% CI (2.0, 8.1)), and maternal primary education (β = 3.5, 95% CI (0.1, 6.8)) were positively associated with MAR scores. School-going adolescents also had significantly higher MAR scores compared to those not attending school (β = 5.3, 95% CI (3.4, 7.3)). Underweight adolescents had lower MAR scores than those with a normal BMI (β = −2.8, 95% CI (−5.4, −0.3)). Lower MAR scores were also observed among adolescents reporting 3–5 days of moderate-to-vigorous physical activity (β = −3.5, 95% CI (−6.7, −0.2)) and those engaging in vigorous physical activity on more than five days per week (β = −3.6, 95% CI (−7.2, −0.1)). Adolescents with fair sleep quality had significantly higher MAR scores (β = 7.3, 95% CI (2.7, 11.9)) compared to those with poor sleep quality. Eating between meals always (β = 5.5, 95% CI (2.9, 8.1)) was associated with higher MAR scores, while the use of bar soap for handwashing was also positively associated with dietary adequacy (β = 6.7, 95% CI (4.4, 8.9)) (Table 3).

In multivariable analysis, adolescents aged 13–15 years continued to demonstrate significantly lower MAR scores compared to those aged 10–12 years (Adjusted β = −3.0, 95% CI (−5.0, −0.9)), while male adolescents had significantly higher MAR scores than females (Adjusted β = 3.3, 95% CI (1.8, 4.8)). Adolescents from the richest wealth quintile had higher MAR scores compared to those from the poorest households (Adjusted β = 5.8, 95% CI (1.8, 9.9)). Underweight adolescents remained significantly associated with lower dietary adequacy (Adjusted β = −2.4, 95% CI (−4.8, −0.1)). Adolescents reporting 3–5 days of moderate-to-vigorous physical activity had lower MAR scores (Adjusted β = −4.0, 95% CI (−7.6, −0.3)). Eating between meals sometimes (Adjusted β = 4.1, 95% CI (1.3, 6.8)) or always (Adjusted β = 6.7, 95% CI (3.9, 9.5)) remained positively associated with MAR scores. Similarly, the use of bar soap for handwashing was independently associated with higher MAR scores compared to households without soap availability (Adjusted β = 5.0, 95% CI (2.0, 7.9)) (Table 3).

## 4. Discussion

This study assessed macro- and micronutrient inadequacies and their factors associated with MAR among adolescents aged 10–19 years in rural Sindh, Pakistan. The mean MAR score is 54.04 out of 100, which reflects an overall poor dietary adequacy, with very high levels of inadequacy observed for energy, calcium, vitamin D, and vitamin A. Male sex, highest wealth quintile, habitual eating between meals, and availability of bar soap were significantly associated with higher dietary adequacy, while underweight and moderate physical activity were significantly associated with lower scores. These findings highlight the complexity of adolescent nutrition in rural Pakistan and the need for targeted interventions addressing socioeconomic and behavioral factors associated with dietary inadequacy.

The high prevalence of nutrient inadequacy observed in this study is consistent with findings from comparable South Asian populations. Because the present study assessed dietary inadequacy rather than biological nutrient status, direct comparisons with biochemical deficiency estimates should be made cautiously. Nevertheless, studies from Pakistan have reported a high prevalence of vitamin D and vitamin A deficiencies among adolescent and young women, with vitamin D deficiency exceeding 81% and vitamin A deficiency approaching 32% [17]. Similarly, studies from rural Bangladesh using the NAR and MAR framework reported inadequate intakes of calcium, vitamin A, and folate in over 73% of adolescent girls, with inadequacy significantly higher among adolescents than adult women [18]. These findings collectively suggest substantial micronutrient vulnerability during adolescence, a period characterized by rapid growth, skeletal development, and reproductive maturation [19]. The comparatively better adequacy observed for protein and iron likely reflects the cereal and legume-based dietary patterns prevalent in rural Sindh, where dal and roti provide modest but measurable contributions to protein and non-haem iron intake [20].

Female adolescents had significantly lower MAR scores than males, reflecting gender-based food allocation practices in rural Pakistani households where boys are commonly prioritized in food distribution, a pattern well documented across South Asian settings [20,21]. The nutritional vulnerability of adolescent girls is particularly concerning given their heightened requirements during puberty and the long-term implications for reproductive health and intergenerational nutrition [21]. Dietary adequacy also declined significantly among early adolescents aged 13–15 years, which may be related to the heightened nutritional demands of the pubertal growth spurt that existing dietary patterns in this setting fail to meet [6]. The non-significant difference observed for the oldest age group may reflect greater food autonomy or stabilization of dietary patterns in late adolescence.

Wealth was an important factor associated with dietary adequacy, and a threshold effect was observed whereby only adolescents in the richest wealth quintile had significantly higher MAR scores compared to the poorest, with no meaningful gradient across intermediate quintiles. This finding is consistent with evidence from rural Pakistan, where wealth quintile was the strongest predictor of dietary diversity, with poverty identified as the most important barrier to accessing nutritious foods and where dietary diversification strategies alone are unlikely to improve diets without addressing purchasing power constraints [22]. The absence of a gradient across intermediate wealth groups likely reflects the overall uniformity of poverty in rural Sindh, where even households above the poorest quintile face limited market access and food purchasing capacity. This is supported by evidence from the same setting where the absence of differences across wealth gradients was attributed to the overall uniformity in poverty, with over 57% of households living below government-defined poverty thresholds [23].

Habitual eating between meals was significantly associated with higher MAR scores, consistent with evidence that greater meal frequency provides more opportunities across the day to meet nutrient requirements. A systematic review similarly demonstrated a positive association between higher meal frequency and better nutritional status and food consumption patterns among adolescents [24]. In this resource-constrained setting, the ability to eat between meals likely also reflects better overall household food access rather than dietary behavior alone. The finding that moderate physical activity was associated with lower dietary adequacy appears counterintuitive but is plausible in this rural context. In rural agricultural households across LMICs, increased energy expenditure associated with physical labor has been identified as an important disconnect between activity levels and nutritional outcomes, where higher caloric demands are not met by available dietary intake [25]. Additionally, in rural settings, physical activity may partly reflect involvement in agricultural or domestic activities; however, occupational activity was not directly measured in this study.

Although the present study primarily identified socioeconomic and behavioral determinants of dietary adequacy, broader food choice factors may also influence adolescents’ dietary patterns. Cultural food preferences and ethnocentric tendencies toward consuming familiar or locally produced foods can shape food selection, particularly in rural communities where traditional diets predominate [26]. Similarly, perceptions regarding the origin of food products and, increasingly, environmental or ecological considerations related to food production may influence dietary choices where food options and consumer awareness permit. However, these constructs were not assessed in the present study, and their relevance in resource-constrained rural Pakistan settings, where food choices are often driven by affordability and availability rather than consumer preference, remains uncertain [26]. Future research should examine how cultural identity, perceptions of locally produced foods, and sustainability-related attitudes interact with socioeconomic constraints to influence adolescent dietary quality.

Underweight adolescents had significantly lower MAR scores, reflecting the co-occurrence of insufficient food intake and poor overall dietary quality. This is consistent with the double burden of malnutrition documented across LMICs, where undernutrition and micronutrient deficiencies frequently coexist, with adolescence identified as a particularly vulnerable period [27]. Although overweight adolescents showed a non-significant trend toward higher MAR, energy excess in LMIC settings frequently coexists with micronutrient deficiency, consistent with the concept of hidden hunger [27]. Beyond nutritional status, the availability of bar soap for handwashing was also significantly associated with higher dietary adequacy. However, this finding should be interpreted cautiously, as the availability of soap is likely to serve as a proxy for broader household characteristics, including socioeconomic status, parental education, living conditions, hygiene practices, and health literacy, rather than representing a direct causal determinant of dietary adequacy. Furthermore, poor diet quality, micronutrient deficiencies, and inadequate hygiene practices have been shown to contribute to a cycle of infection and undernutrition among adolescents in Pakistan [28], with evidence from South Asia similarly confirming that non-use of soap is significantly associated with poorer nutritional status among adolescent girls [29], further underscoring the need for integrated nutrition and WASH interventions in this setting.

Several strengths of this study deserve mention. The large representative community-based sample drawn from a rural underserved population in Pakistan enhances the representativeness of findings for similar LMIC settings. Validated tools were used across all exposures and outcomes, and the complex survey design with appropriate weighting and clustering adjustments ensured methodologically rigorous estimates. Notably, the use of MAR as a continuous composite measure of dietary adequacy offers a more holistic assessment of overall diet quality than binary inadequacy classifications for individual nutrients, avoiding the statistical limitations associated with very high prevalence estimates of nutrient inadequacy. Furthermore, multivariable modeling controlled for a comprehensive range of sociodemographic, behavioral, and psychosocial factors, strengthening the validity of identified associations.

This study has several limitations that should be considered when interpreting the findings. Dietary intake was assessed using a single 24-h dietary recall at one time point, which may not reflect participants’ usual dietary intake because of day-to-day variation in food consumption. Consequently, the reported prevalence of nutrient inadequacy should be interpreted cautiously, as it may not accurately reflect habitual intake at the population level. In addition, nutrient adequacy ratios (NARs) and the mean adequacy ratio (MAR) were calculated using absolute nutrient intakes without adjustment for total energy intake. Therefore, MAR values may reflect both overall food consumption and nutrient adequacy rather than nutrient density alone, and adolescents with lower energy intake may have lower MAR scores partly because of reduced overall food intake. No biochemical confirmation of nutritional status through blood biomarkers was available, meaning adequacy classification relied solely on self-reported dietary intake [30]. Future studies should complement dietary assessment with objective biochemical, hematological, immunological, oxidative stress, and adipokine biomarkers to provide a more comprehensive assessment of nutritional status and improve the identification of clinical and subclinical malnutrition, thereby strengthening the clinical interpretation of dietary inadequacy [31]. Depression was assessed using the Beck Depression Inventory (BDI), which has been primarily validated among adolescents aged ≥13 years; therefore, findings related to depressive symptoms among participants aged 10–12 years should be interpreted with caution. The cross-sectional design precludes causal inference between identified determinants and dietary adequacy. Although nutrient intake was estimated using the harmonized MAL-ED Pakistan Food Composition Table, some measurement error may remain because the database incorporates nutrient values from multiple international sources, which may differ in food composition and analytical methods. In addition, anthropometric measurements were conducted during household visits at varying times of the day according to participant availability rather than at a standardized time, which may have introduced minor measurement variability, particularly for body weight. Finally, the single-stratum survey design limits the generalizability of the findings beyond rural Sindh, Pakistan.

These findings have significant implications for public health policies and nutrition programs targeting adolescent dietary adequacy in rural Pakistan. School health programs should incorporate nutrition education emphasizing dietary diversity, food group consumption, and age-specific nutrient requirements, equipping adolescents with the knowledge and skills to make informed dietary choices. Parent-focused campaigns can foster household environments that promote equitable food distribution and prioritize the nutritional needs of adolescent girls. Given that a considerable proportion of adolescents in rural Sindh are out of school, community-wide initiatives involving local healthcare providers, NGOs, and community leaders are essential to ensure broad reach. Interventions addressing dietary inadequacy could be delivered through community health workers, mobile clinics, and community-based platforms to maximize accessibility in underserved areas. At the policy level, the development of nationally adapted dietary guidelines for adolescents and systematic integration of nutrition assessment into existing rural health programs would enable early identification of at-risk adolescents and timely linkage to nutrition support services. These efforts align with Sustainable Development Goal 2, which aims to end hunger and achieve food security, and Sustainable Development Goal 3, which focuses on ensuring healthy lives and promoting wellbeing for all ages.

## 5. Conclusions

The study provides comprehensive evidence of the substantial burden of dietary inadequacy among adolescents in rural Pakistan, where overall diet quality was poor and inadequacy was particularly pronounced for several key micronutrients and energy intake. Dietary adequacy was associated with a complex set of socioeconomic, behavioral, and nutritional factors, with wealth, gender, eating patterns, physical activity, BMI, and hygiene practices all contributing independently to overall diet quality. These findings underscore that dietary inadequacy in this population is not a simple problem of insufficient food supply but reflects deeply entrenched structural inequities that require coordinated, multi-sectoral responses. Addressing adolescent undernutrition in rural Pakistan demands targeted gender-sensitive interventions, strengthened food security policies, and context-specific nutrition programs delivered through schools and community platforms. Investment in adolescent nutrition during this critical period of growth and development is essential not only for immediate health outcomes but also for breaking the intergenerational cycle of malnutrition in countries like Pakistan.

## Figures and Tables

**Table 1:** Sociodemographic, household and nutritional characteristics and lifestyle factors of study participants (N = 1132).

Variable	n (%)
**Age group (years)**	
10–12	351 (31.0)
13–15	410 (36.2)
16–19	371 (32.8)
**Gender**	
Female	577 (51.0)
Male	555 (49.0)
**Household members**	
≤5	404 (35.7)
>5	728 (64.3)
**Sanitation facility**	
Unimproved	816 (72.1)
Improved	316 (27.9)
**Father’s education**	
Never attended	631 (55.7)
Primary	251 (22.2)
Secondary	140 (12.4)
College/University	110 (9.7)
**Mother’s education**	
Uneducated	1005 (88.8)
Primary	102 (9.0)
Secondary	19 (1.7)
College and higher	6 (0.5)
**Currently going to school**	
Not attending school	711 (62.8)
School going	421 (37.2)
**Marital status**	
Yes	56 (5.0)
No	1076 (95.0)
**Food insecurity status**	
Food insecure	976 (86.2)
Food secure	156 (13.8)
**Household dietary diversity**	
Low (≤3 food groups)	82 (7.2)
Medium (4–5 food groups)	754 (66.6)
High (≥6 food groups)	296 (26.1)
**BMI**	
Normal	880 (77.7)
Underweight	207 (18.3)
Overweight	45 (4.0)
**Physical activity category**	
<3 days inactive	890 (78.6)
3–5 days MVPA	106 (9.4)
>5 days VPA	136 (12.0)
**Sleep quality**	
Bad	62 (5.5)
Good	820 (72.4)
Fairly good	250 (22.1)
**Depression**	
Yes	121 (10.7)
No	1011 (89.3)
**Eating between meals**	
Never	391 (35.0)
Sometimes	466 (41.7)
Always	261 (23.3)
**Handwashing material**	
No soap available	278 (24.6)
Bar soap	735 (64.9)
Ash/Mud	68 (6.0)
Others	51 (4.5)
**Child labor**	
Yes	401 (35.4)
No	731 (64.6)
**Working hours/day**	
0–3	128 (11.3)
4–6	177 (15.6)
7–12	96 (8.5)
Not working	731 (64.6)

**Table 2:** Mean dietary intake and inadequacy of macro- and micronutrients (N = 1132).

Nutrients	Mean (SD)	Adequate	Inadequate
Macronutrients			
Energy (kcal) (EER: 1925–2820 kcal/day)	1149.56 (531.54)	39 (3.4%)	1093 (96.6%)
Protein (g) (EAR: 0.66–0.76 g/kg/day) *	35.70 (14.20)	746 (66.1%)	383 (33.9%)
Total Fat (g) (AMDR: 20–35% of total energy)	40.72 (27.36)	803 (70.9%)	329 (29.1%)
Fiber (g) (AI: 25–38 g/day) ^a^	16.37 (14.75)	192 (17.0%)	940 (83.0%)
Carbohydrate (g) (EAR: ≥100 g/day)	260.39 (88.81)	1118 (98.8%)	14 (1.2%)
Vitamins			
Vitamin A (Retinol) (mcg) (EAR: 420–630 µg RAE/day)	155.74 (94.44)	16 (1.4%)	1116 (98.6%)
Vitamin B1 Thiamin (mg) (EAR: 0.7–1.0 mg/day)	0.73 (0.53)	787 (69.5%)	345 (30.5%)
Vitamin B2 Riboflavin (mg) (EAR: 0.8–1.1 mg/day)	0.76 (0.44)	794 (70.1%)	338 (29.9%)
Vitamin B3 Niacin (mg) (EAR: 9–12 mg/day)	10.14 (6.00)	726 (64.1%)	406 (35.9%)
Vitamin B9 Folate (mcg) (EAR: 250–330 µg/day)	124.19 (83.22)	62 (5.5%)	1070 (94.5%)
Vitamin B6 Pyridoxine (mg) (EAR: 0.8–1.1 mg/day) *	0.81 (0.43)	368 (33.6%)	728 (66.4%)
Vitamin C (mg) (EAR: 39–75 mg/day)	15.25 (11.92)	113 (10.0%)	1019 (90.0%)
Vitamin D (mcg) (EAR: 10 µg/day)	0.39 (0.42)	11 (1.0%)	1121 (99.0%)
Vitamin E (mg) (EAR: 9–12 mg/day)	2.97 (2.91)	19 (1.7%)	1113 (98.3%)
Minerals			
Calcium (mg) (EAR: 800–1100 mg/day)	336.23 (192.01)	7 (0.6%)	1125 (99.4%)
Iron (mg) (EAR: 5.7–8.9 mg/day)	6.05 (3.32)	757 (66.9%)	375 (33.1%)
Magnesium (mg) (EAR: 200–340 mg/day)	164.50 (89.19)	168 (14.8%)	964 (85.2%)
Phosphorus (mg) (EAR: 580–1055 mg/day)	688.66 (290.23)	144 (12.7%)	988 (87.3%)
Potassium (mg) (AI: 2300–3400 mg/day) ^a^	1172.61 (480.34)	16 (1.4%)	1116 (98.6%)
Sodium (mg) (AI: 1000–1500 mg/day) ^a^	869.43 (498.36)	184 (16.3%)	948 (83.7%)
Zinc (mg) (EAR: 6.8–9.4 mg/day)	5.49 (2.32)	187 (16.5%)	945 (83.5%)

* Extreme outliers for vitamin B6 intake were excluded prior to analysis to minimize the influence of implausible values on descriptive statistics. ^a^ Dietary fiber, potassium, and sodium are based on Adequate Intake (AI) values rather than Estimated Average Requirements (EARs). Therefore, percentages shown represent the proportion of participants meeting or falling below the AI recommendation and should not be interpreted as estimates of the prevalence of inadequate intake.

**Table 3:** Factors associated with MAR among adolescents aged 10–19 years in TMK (N = 1132).

Category	MAR (Mean ± SD)	Crude β (95% CI)	Adjusted β (95% CI)
**Overall**	54.0 ± 16.5	--	--
**Age group (years)**			
10–12	55.8 ± 15.9	Reference	Reference
13–15	52.6 ± 17.1	−2.7 (−4.7, −0.7) *	−3.0 (−5.0, −0.9) *
16–19	53.6 ± 16.4	−1.9 (−4.5, 0.7)	−1.9 (−4.8, 1.0)
**Gender**			
Female	52.5 ± 16.2	Reference	Reference
Male	55.5 ± 16.8	3.0 (1.1, 4.9) *	3.3 (1.8, 4.8) *
**Wealth quintile**			
Poorest	50.0 ± 17.7	Reference	Reference
Poor	51.6 ± 16.0	2.5 (−0.7, 5.8)	2.3 (−0.5, 5.2)
Middle	53.9 ± 14.7	4.1 (0.6, 7.5) *	2.9 (−0.4, 6.2)
Rich	53.1 ± 15.7	3.0 (−0.4, 6.5)	0.0 (−3.3, 3.3)
Richest	61.3 ± 16.2	11.9 (8.5, 15.3) *	5.8 (1.8, 9.9) *
**Household members**			
≤5	54.4 ± 15.7	Reference	--
>5	53.7 ± 17.0	−0.4 (−2.7, 1.9)	--
**Sanitation facility**			
Unimproved	52.7 ± 16.5	Reference	--
Improved	56.8 ± 16.3	4.2 (2.0, 6.3) *	--
**Father’s education**			
Never attended	52.3 ± 15.9	Reference	Reference
Primary	53.2 ± 15.6	0.6 (−1.8, 3.1)	−1.3 (−3.7, 1.1)
Secondary	57.3 ± 17.6	5.0 (2.0, 8.1) *	3.2 (0.0, 6.3)
College/University	60.3 ± 18.9	8.0 (3.6, 12.3) *	1.6 (−2.3, 5.4)
**Mother’s education**			
Uneducated	53.4 ± 16.4	Reference	--
Primary	56.9 ± 17.3	3.5 (0.1, 6.8) *	--
Secondary	59.2 ± 18.7	5.8 (−1.7, 13.3)	--
College and higher	62.4 ± 17.6	9.0 (−4.3, 22.2)	--
**Currently going to school**			
Not attending school	51.9 ± 16.3	Reference	--
School going	57.2 ± 16.5	5.3 (3.4, 7.3) *	--
**Marital status**			
Yes	50.9 ± 18.2	Reference	--
No	54.1 ± 16.5	3.4 (−1.9, 8.8)	--
**Food insecurity status**			
Food insecure	53.2 ± 16.2	−7.6 (−10.7, −4.6) *	--
Food secure	59.8 ± 17.1	Reference	--
**Household dietary diversity**			
Low (≤3 food groups)	60.1 ± 18.4	Reference	--
Medium (4–5 food groups)	54.1 ± 16.5	−6.2 (−10.1, −2.3) *	--
High (≥6 food groups)	51.8 ± 15.7	−9.1 (−13.2, −4.9) *	--
**BMI**			
Normal	54.4 ± 16.4	Reference	Reference
Underweight	50.9 ± 16.6	−2.8 (−5.4, −0.3) *	−2.4 (−4.8, −0.1) *
Overweight	58.6 ± 16.5	5.1 (0.0, 10.2)	3.7 (−1.0, 8.5)
**Physical activity category**			
<3 days inactive	54.6 ± 16.7	Reference	Reference
3–5 days MVPA	51.8 ± 14.6	−3.5 (−6.7, −0.2) *	−4.0 (−7.6, −0.3) *
>5 days VPA	51.0 ± 16.7	−3.6 (−7.2, −0.1) *	−2.7 (−6.2, 0.8)
**Sleep quality**			
Fairly Bad	48.3 ± 18.9	Reference	--
Fairly good	55.7 ± 16.4	7.3 (2.7, 11.9) *	--
Very good	53.8 ± 16.3	5.4 (1.2, 9.7) *	--
**Depression**			
Yes	49.2 ± 16.5	−5.2 (−8.5, −1.9) *	--
No	54.4 ± 16.4	Reference	--
**Eating between meals**			
Never	51.5 ± 16.9	Reference	Reference
Sometimes	54.1 ± 16.1	2.4 (−0.4, 5.2)	4.1 (1.3, 6.8) *
Always	57.2 ± 16.2	5.5 (2.9, 8.1) *	6.7 (3.9, 9.5) *
**Handwashing material**			
No soap available	49.2 ± 16.3	Reference	Reference
Bar soap	55.9 ± 16.4	6.7 (4.4, 8.9) *	5.0 (2.0, 7.9) *
Ash/Mud	51.3 ± 13.9	2.1 (−2.2, 6.4)	0.4 (−4.4, 5.2)
Others	53.3 ± 19.0	4.0 (−1.6, 9.6)	−2.4 (−10.3, 5.5)
**Child labor**			
Yes	53.3 ± 16.5	Reference	--
No	54.3 ± 16.6	1.5 (−1.0, 3.9)	--
**Working hours/day**			
0–3	54.1 ± 16.0	Reference	--
4–6	53.6 ± 16.7	−1.0 (−4.4, 2.3)	--
7–12	51.7 ± 16.9	−2.2 (−6.5, 2.1)	--
Not working	54.3 ± 16.6	0.5 (−2.8, 3.7)	--

* *p* < 0.05.

## Data Availability

Data available on request from the authors.

## Supplementary Materials

The following supporting information can be downloaded at: https://www.mdpi.com/article/10.3390/nu18152438/s1. Figure S1. Diagnostic plots used to assess the assumptions of the multivariable linear regression model evaluating factors associated with mean adequacy ratio (MAR-100). (A) Histogram of standardized residuals with superimposed normal density curve. (B) Normal quantile–quantile (Q–Q) plot of standardized residuals. (C) Normal probability–probability (P–P) plot of standardized residuals. (D) Standardized residuals versus fitted values for assessment of linearity and homoscedasticity. (E) Cook's distance plotted against observation number for identification of potentially influential observations.
