# Supplementary material for: Dietary Adequacy and Its Associated Factors Among Adolescents in a Rural District of Sindh
Source: Nutrients. 2026 Jul 26;18(15):2438. doi: 10.3390/nu18152438 (PMC13468357; doi:10.3390/nu18152438)
Supplement: Supplementary file 1 [file nutrients-18-02438-s001.zip › nutrients-4427446-supplementary.pdf]

Supplementary File

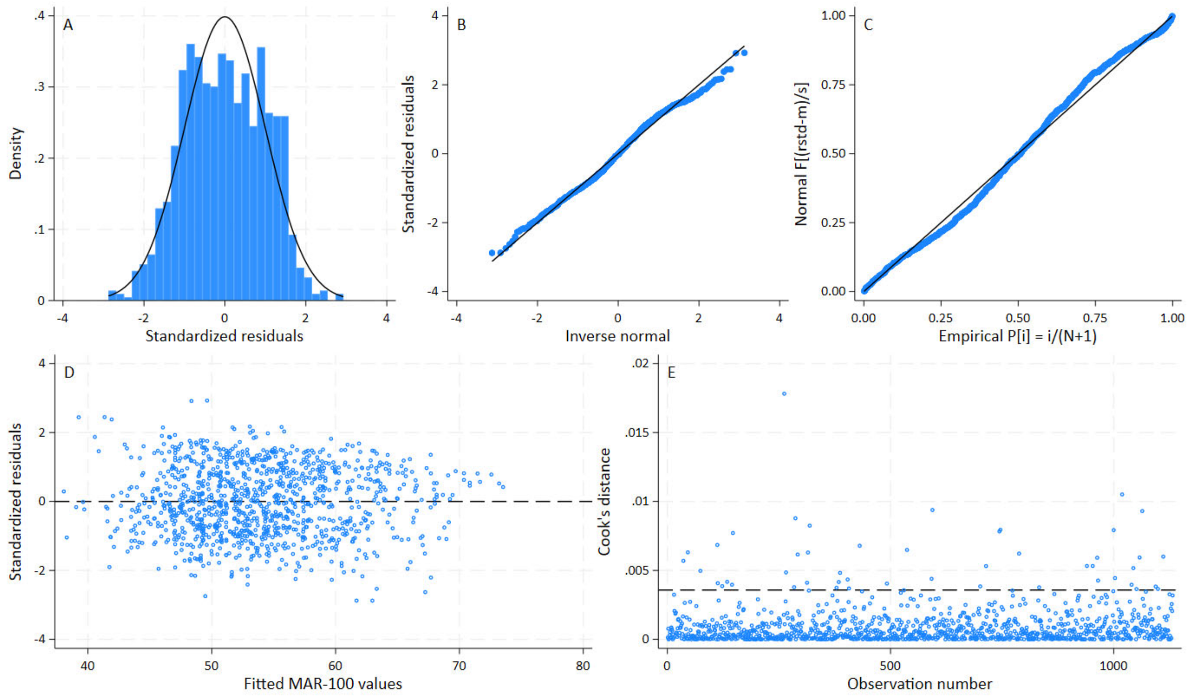

Supplementary Figure S1. Diagnostic plots used to assess the assumptions of the multivariable linear regression model evaluating factors associated with mean adequacy ratio (MAR-100). (A) Histogram of standardized residuals with superimposed normal density curve. (B) Normal quantile–quantile (Q–Q) plot of standardized residuals. (C) Normal probability–probability (P–P) plot of standardized residuals. (D) Standardized residuals versus fitted values for assessment of linearity and homoscedasticity. (E) Cook's distance plotted against observation number for identification of potentially influential observations.
